# Trends in Glucocerebrosides Research: A Systematic Review

**DOI:** 10.3389/fphys.2020.558090

**Published:** 2020-10-29

**Authors:** Mazarine Desplanque, Marie-Amandine Bonte, Bernard Gressier, David Devos, Marie-Christine Chartier-Harlin, Karim Belarbi

**Affiliations:** ^1^Univ. Lille, Inserm, CHU-Lille, Lille Neuroscience and Cognition, Lille, France; ^2^Département de Pharmacologie de la Faculté de Pharmacie, Univ. Lille, Lille, France; ^3^Département de Pharmacologie Médicale, I-SITE ULNE, LiCEND, Lille, France

**Keywords:** cancer drug resistance, gangliosides, gaucher disease, glucocerebrosides, glucosylceramides, lipids, Parkinson Disease, sphingolipids

## Abstract

Glucocerebrosides are sphingolipid components of cell membranes that intervene in numerous cell biological processes and signaling pathways and that deregulation is implicated in human diseases such as Gaucher disease and Parkinson's disease. In the present study, we conducted a systematic review using document co-citation analysis, clustering and visualization tools to explore the trends and knowledge structure of glucocerebrosides research as indexed in the Science Citation Index Expanded database (1956—present). A co-citation network of 5,324 publications related to glucocerebrosides was constructed. The analysis of emerging categories and keywords suggested a growth of research related to neurosciences over the last decade. We identified ten major areas of research (e.g., clusters) that developed over time, from the oldest (i.e., on *glucocerebrosidase protein* or *molecular analysis of the GBA gene*) to the most recent ones (i.e., on *drug resistance in cancer, pharmacological chaperones*, or *Parkinson's disease*). We provided for each cluster the most cited publications and a description of their intellectual content. We moreover identified emerging trends in glucocerebrosides research by detecting the surges in the rate of publication citations in the most recent years. In conclusion, this study helps to apprehend the most significant lines of research on glucocerebrosides. This should strengthen the connections between scientific communities studying glycosphingolipids to facilitate advances, especially for the most recent researches on cancer drug resistance and Parkinson's disease.

## Introduction

**Figure 1:**
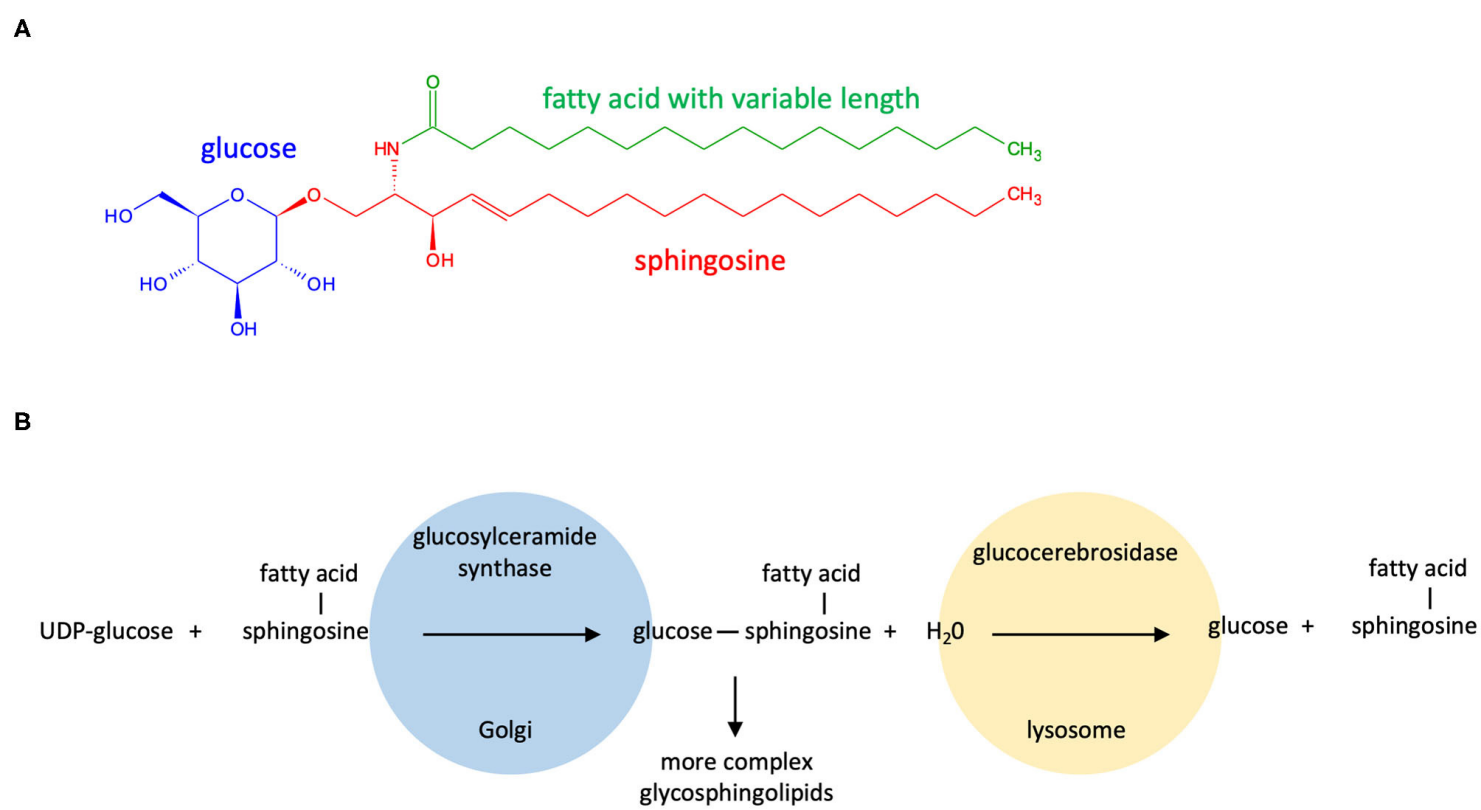
Structure and metabolism of glucocerebrosides. **(A)** Glucocerebrosides are composed of a sphingosine, a fatty acid chain (these two forming a ceramide) and a single glucose residue. The fatty acid attached to the sphingosine may vary in chain length and in degree of unsaturation; **(B)** Glucocerebrosides are synthesized in the Golgi apparatus by the rate-limiting enzyme glucocerebroside synthase (UGCG; located on locus 9q31; also named glucosylceramide synthase) that uses UDP-glucose as the glycosyl donor. The catabolism of glucocerebrosides is primarily initiated in the lysosomes by the action of the lysosomal glucocerebrosidase (encoded by the *GBA* gene located on locus 1q21). Glucocerebrosides can serve as precursors for the production of more complex glycosphingolipids such as lactosylceramide and gangliosides by sequential addition of sugars and other chemical groups.

Glucocerebrosides (also referred to as glucosylceramides) are components of cell membranes in organisms from bacteria to humans. They are composed of a sphingosine, a fatty acid chain (these two forming a ceramide) and a glucose moiety and are found in all mammalian tissues being particularly abundant in the brain. In 1934, the French gynecologist Henriette Aghion identified glucocerebrosides as the lipids that accumulate in the enlarged spleen and liver of patients with Gaucher disease (ORPHA355), a lysosomal storage disorder with three clinical types: non-neuropathic (type 1); acute neuropathic (type 2); and chronic neuropathic (type 3) (Aghion, 1934; Stirnemann et al., 2017). This eventually led to the discovery that Gaucher disease is due to loss-of-function mutations present on both alleles of the *GBA* gene encoding glucocerebrosidase (also named acid beta-glucosylceramidase or beta-glucocerebrosidase), a 497-residue enzyme that breaks down glucocerebrosides into glucose and ceramides inside lysosomes (Brady et al., 1965a,b; Figure 1).

Today, the biological and medical importance of glucocerebrosides is well established. As biologically active components of cell membranes, glucocerebrosides and their derived glycosphingolipids intervene in several biological processes including embryogenesis (Yamashita et al., 1999), cell polarity (Zhang et al., 2011), cell adhesion and migration (Proia, 2003; Furukawa et al., 2004), and energy homeostasis (Nordstrom et al., 2013) and also regulate the activity of plasma membrane proteins, including protein tyrosine kinases (Suzuki, 2012). Alterations in the metabolism of glucocerebrosides have also been implicated in many human diseases besides Gaucher disease including cardiovascular disease (Edsfeldt et al., 2016), diabetes (Chavez et al., 2014), skin disorders (Feingold and Elias, 2014), cancer (Ogretmen and Hannun, 2004), or in the expression of multidrug resistance (Gouaze et al., 2005). Glucocerebrosides have also taken center stage in the field of neurodegeneration since the presence of *GBA* mutation on a single allele has been associated to increased risks for Parkinson's disease (Sidransky et al., 2009) and dementia with Lewy bodies (Nalls et al., 2013), the two most common dementing neurodegenerative diseases after Alzheimer's disease.

Glucocerebrosides-related literature has been published over years across many research areas and diseases. However, to the best of our knowledge, a systematic analysis of the scientific literature in glucocerebrosides research has not been conducted to date. Science mapping and visualization help to explore the scientific knowledge. In particular, document co-citation analysis enables the identification of relevant literature and scholarly communities that may be overlooked in standard approaches to literature searching, as well as their societal influence (Trujillo and Long, 2018; Zeng et al., 2019). In the present study, document co-citation analysis, clustering and visualization tools were used to systematically review the research relating to glucocerebrosides from publications retrieved on the Science Citation Index Expanded of the Web of Science Core Collection online database (1956–present). Results are presented to provide an unbiased picture of the literature and of the scholarly communities involved in this research and to identify its new developments and frontiers.

## Materials and Methods

### Source of the Data and Search Strategy

The search was performed on the Science Citation Index-Expanded (SCI-E) of the Web of Science Core Collection online database (Thomson Reuters) hosted by Clarivate Analytics. All electronic searches were conducted on a single day, July 3, 2019, to avoid changes in citation rate as much as possible. The string “TOPIC:(glucocerebrosid^*^ OR glucosylceramid^*^) *AND* Language:(English)” was used to retrieve any documents published from 1956 to 2019 mentioning for example “glucocerebrosides,” “glucosylceramides,” “glucosylceramide synthase,” or “glucocerebrosidase” in their titles, abstracts, author keywords, or KeyWord Plus (keywords unique to Web of Science that consist of words and phrases harvested from the titles of the cited articles). Retracted publications were excluded. Bibliometric data were extracted including titles, author information, abstracts, keywords, source, publication year, Web of Science category of the publication and cited references. The data were downloaded in plain text and imported in CiteSpace 5.5.R2 (64-bit) (Synnestvedt et al., 2005) for analysis.

### Subject Category and Keyword Analysis

Every record in Web of Science Core Collection is assigned to at least one subject category of its source publication. CiteSpace was used to generate and visualize networks of the journal-based Web of Science Subject Categories assigned to the publications in our dataset. Burst detection was applied to detect subject categories or keywords that had a surge of their appearance/citation for a specific period of time.

### Document Co-citation Analysis

A document co-citation network represents a network of references that have been co-cited by a set of publications. Briefly, if two articles are both cited as references in another article, then these two papers have a co-citation relationship (Small, 1973). Citespace was used to generate document co-citation networks derived from the 50 most cited articles published in every single year y as Document co-citation analysis (DCA[y]). The time series of these individual DCAs were then integrated for all the period to produce a synthesized network. The synthetized network was divided into a number of clusters of co-cited references so that tightly coupled references were within the same clusters and loosely connected references were in different clusters. Log-likelihood ratio test method was used to label these clusters with terms extracted from the titles of the most representative articles for each cluster. Betweenness centrality of publications were calculated in an attempt to identify those that bridge two or more clusters. Burst detection was applied to publications to detect those cited at an increasingly faster rate during a period of time. Sigma, introduced by Chen and coworkers in 2009 and defined as (*centrality*+1)^burstness^, was used as a measure of scientific novelty such that the brokerage mechanism plays more prominent role than the rate of recognition by peers (Chen et al., 2009).

## Results

### Publication Outputs

A total of 5,324 publications met the search criteria. These include 3,999 research articles (75.11%), 563 reviews (10.57%), and 511 meeting abstracts (9.59%). According to the analysis of the Web of Science database, the global h-index was 153 with an average citation per item of 31.19.

### Subject Categories Involved in Glucocerebrosides Research

**Figure 2:**
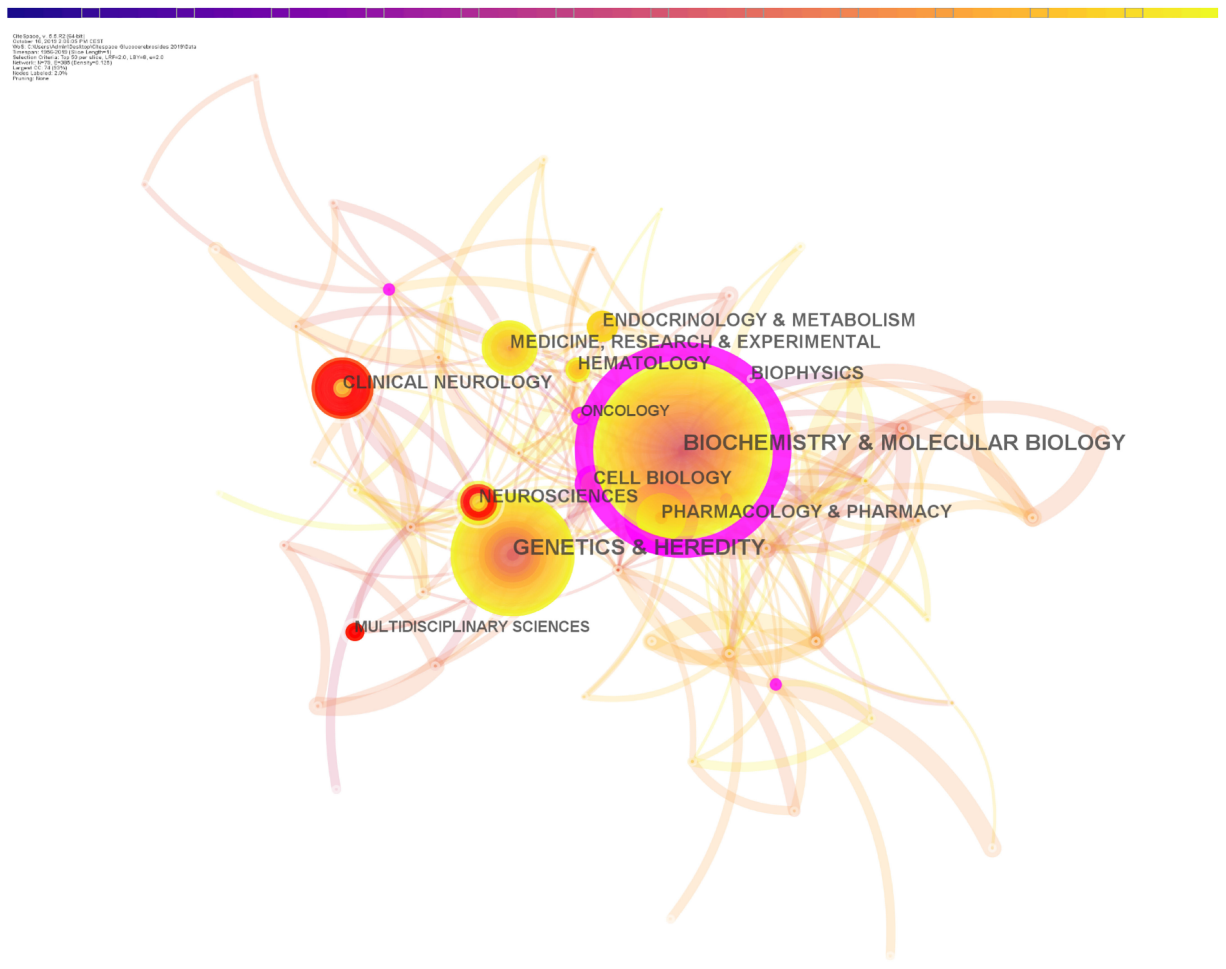
Major categories involved in glucocerebrosides research. In this representation, nodes represent categories and edges represent instances of co-occurrence. A larger radius corresponds to a higher occurrence (log transformed). The thickness of the edges is proportional to the co-occurrence strength. Categories with burst at a certain time are represented with a red ring. Clinical neurology and Neuroscience, with some of its citation rings in red, belong to categories in which the number of articles has increased rapidly.

Each publication indexed in the Web of Science is associated to one or more journal-based subject categories. In our dataset, a total of 68 unique subject categories were found. The most represented categories were: (1) Biochemistry & Molecular Biology (1,534 publications); (2) Genetics & Heredity (742 publications); (3) Medicine, Research & Experimental (519 publications); (4) Clinical neurology (436 publications); (5) Cell Biology (410 publications); (6) Hematology (380 publications); (7) Neuroscience (362 publications); (8) Pharmacology and Pharmacy (358 publications); (9) Biophysics (317 publications); (10) Endocrinology and metabolism (315 publications). Figure 2 shows a network of these Web of Science categories largely assigned to the publications in our dataset. Burstness analysis revealed that the categories Clinical Neurology (25.45) and Neuroscience (37.46) showed very strong bursts starting in 2012 and 2016, respectively (Table 1).

**Table 1:** Web of Science categories with strong occurrence burst in descending order of burst strength.

**Category**	**Count**	**Burstness (period)**	**Range (1956-2019)**
Biochemistry & Molecular biology	1534	63.10 (1965-1992)	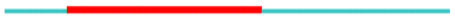
Neurosciences	362	37.46 (2015-2019)	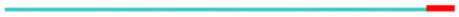
Biophysics	317	36.79 (1973-1993)	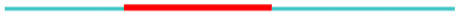
Clinical neurology	436	25.45 (2012-2019)	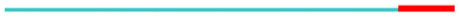
Hematology	380	21.93 (1993-2001)	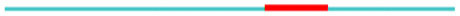

*Red line segments indicate the time slices in which categories bursts, that is, rapid increases of citation counts, are detected*.

### Analysis of Keywords

Publications in the dataset are assigned keywords. The 300 keywords that occurred the most in the 5324 publications or our dataset were extracted and were analyzed for their burstness. The keywords “glucocerebrosidase mutation” (2012, 2019), “Parkinson's disease” (2013, 2019), and “alpha-synuclein” (2014, 2019) were among those with the highest burstness and their abrupt increase in citation all occur in the last decade (Table 2).

**Table 2:** Top 5 keywords with the strongest occurrence burst.

**Keyword**	**Burstness (period)**	**Range (1956-2019)**
Parkinson's disease	66.09 (2013-2019)	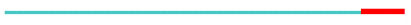
Glucocerebrosidase mutation	40.84 (2012-2019)	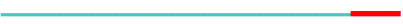
Human glucocerebrosidase gene	37.59 (1991-2002)	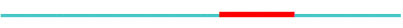
Alpha synuclein	35.03 (2014-2019)	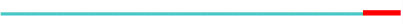
Macrophage targeted glucocerebrosidase	25.46 (1994-2004)	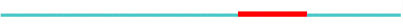

*Red line segments indicate the time slices in which keywords bursts are detected*.

### Co-citation Network Analysis

**Figure 3:**
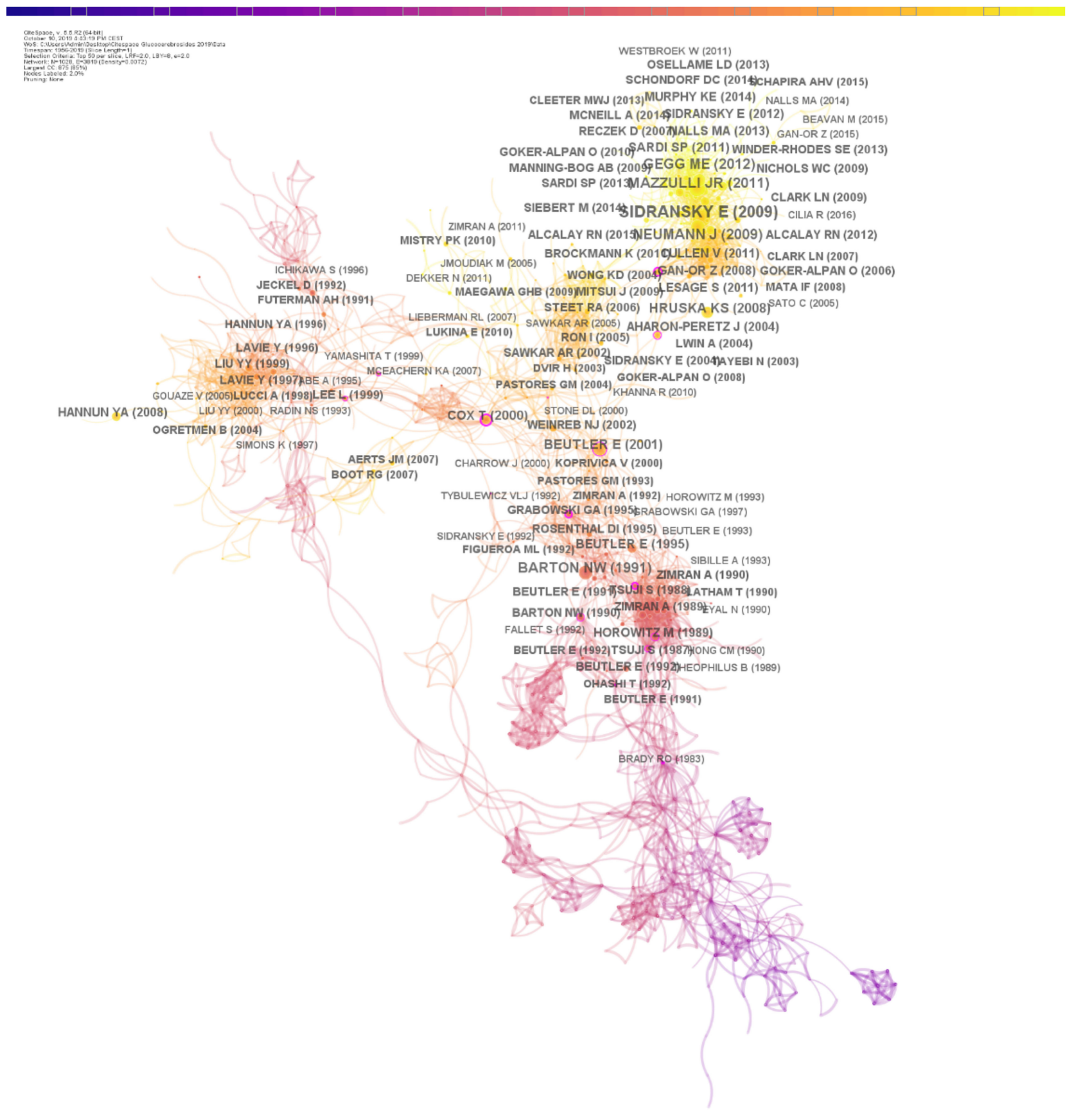
Co-citation network analysis. Co-citation map of references from publications on glucocerebrosides research published between 1956 and 2019. In this representation, nodes represent cited references and edges represent instances of co-citation. A larger radius corresponds to a higher citation in the co-citation network. The thickness of the edges is proportional to the co-citation strength. The color of the lines indicates the time period in which the co-citations first occurred. Nodes with a betweenness centrality >0.1 (e.g., nodes linked with over 10% of the nodes in the whole network) are represented with a purple circle.

Figure 3 shows the synthesized co-citation network for glucocerebrosides research publications. In this representation, nodes represent cited references. The radius of a node increases with its frequency of citations within the network (log transformed). Edges represent instances of co-citation and their thicknesses are proportional to the number of times that two documents are jointly cited. Nodes with a betweenness centrality >0.1 (e.g., nodes linked with over 10% of the nodes in the whole network) are represented with a purple ring. We identified most cited publications within the network (showing the largest radii) as focusing on the causal association between glucocerebrosidase mutations and Parkinson's disease (Neumann et al., 2009; Sidransky et al., 2009), glucocerebrosidase activity deficiency in Parkinson's disease (Gegg et al., 2012), the interaction between glucocerebrosidase and alpha-synuclein (e.g., the protein that accumulates in neurons in Parkinson's disease) (Mazzulli et al., 2011), enzyme replacement therapy in Gaucher disease (Barton et al., 1991), Gaucher disease physiopathology (Beutler and Grabowski, 2001), and glucocerebrosidase mutations in Gaucher disease (Hruska et al., 2008). Publications with the highest betweenness centrality focus on the pathophysiology of Gaucher disease (Brady and Barranger, 1983; Wong et al., 2004), report the heterogeneity of mutations in the *GBA* gene of Gaucher disease patients (Latham et al., 1991) or characterize the links between glucocerebroside metabolism and epidermis (Holleran et al., 1994; Marsh et al., 1995). These latest publications likely bridge two or more underlying lines of research and may be important from different disciplinary perspectives.

### Clustering Analysis of Glucocerebrosides Co-citation Network

**Figure 4:**
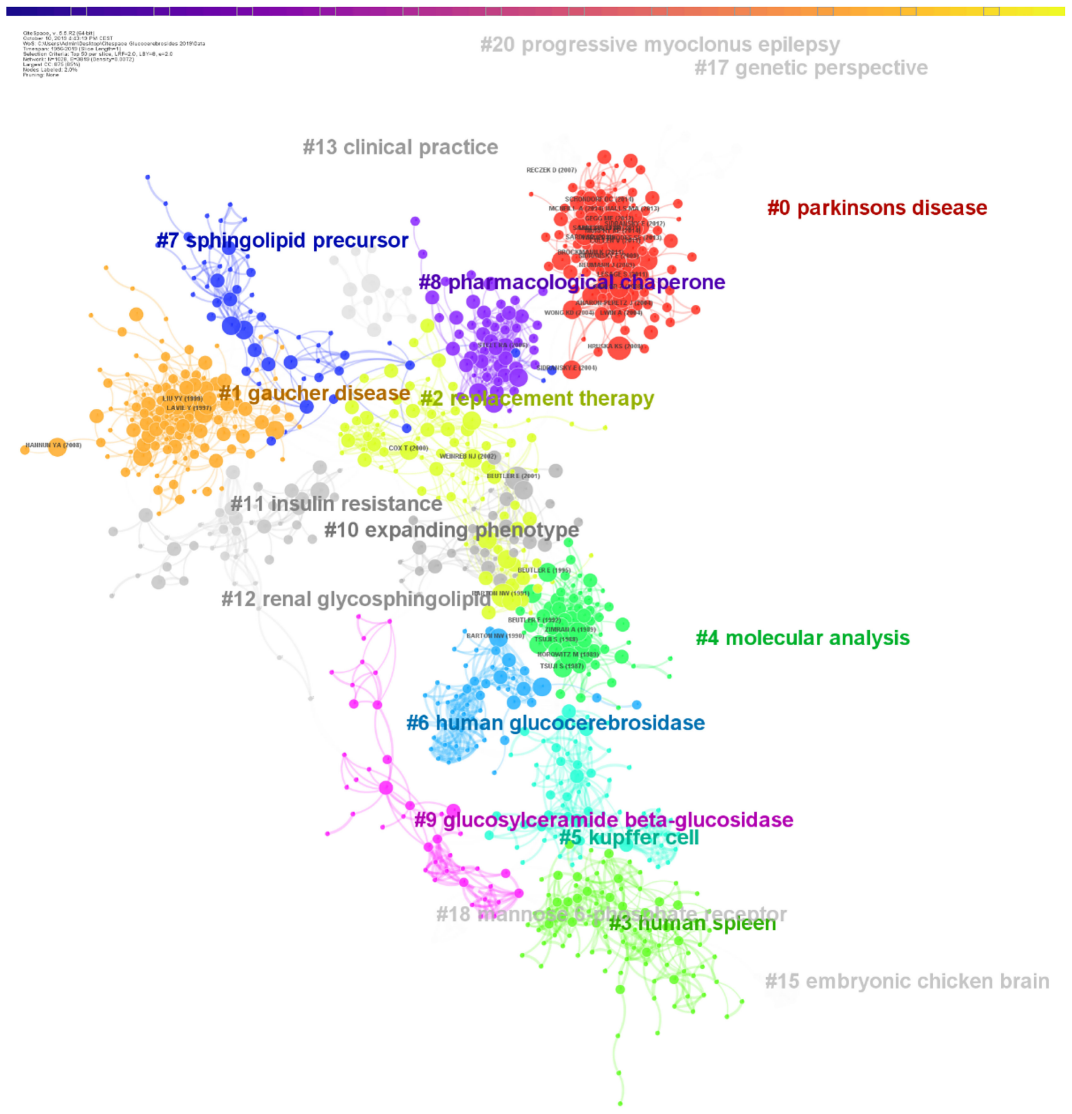
Clustering analysis of the co-citation network. Clusters are labeled by terms extracted from the titles of their most representative articles. For clarity, each cluster has its nodes and label of a unique color. Small clusters may be hidden due to the graphical representation. The youngest cluster is the Cluster #0 (Parkinson's disease) in the higher right corner of the visualization.

The co-citation network for glucocerebrosides research was divided into clusters, so that references that are tightly connected are within the same clusters, but those loosely connected are in different clusters. The modularity Q score was higher than 0.5 (0.8423) indicating that the network was reasonably divided into loosely coupled clusters. Each cluster was labeled by words in the titles of citing publications of the cluster. The automatically chosen cluster label, size and average year of publication of the ten major clusters by their size are summarized in Table 3. The visualization of the network divided into distinct co-citation clusters is seen in Figure 4.

**Table 3:** Major clusters of co-cited references.

**Cluster ID**	**Label**	**Size**	**Mean (year)**
#0	Parkinson's disease	122	2011
#1	Gaucher disease	117	1999
#2	Replacement therapy	94	1999
#3	Human spleen	93	1976
#4	Molecular analysis	68	1991
#5	Kupffer cell	67	1982
#6	Human glucocerebrosidase	53	1988
#7	Sphingolipid precursor	53	1993
#8	Pharmacological chaperone	52	2006
#9	Glucosylceramide beta-glucosidase	41	1987

*Clusters are referred in terms of the labels selected by log-likelihood ratio test method*.

### Intellectual Base (Cited References) of the Ten Major Clusters

The cited publications of a cluster define its intellectual base. Table 4 shows the title and main conclusion of the most cited publication for each of the ten largest clusters. In addition, we provide in Supplementary Table 1 the list of the 10 most-cited members of these clusters as well as their structural, temporal, and saliency metrics such as citation count, betweenness centrality, citation burstness and sigma that can be considered as a measure of scientific novelty (Chen et al., 2009).

**Table 4:** Most frequently cited reference for each of the ten largest document co-citation clusters in the co-citation network.

**Cluster**	**Node's name**	**Title**	**Main conclusion**	**References**
Cluster #0 (Parkinson's disease)	SIDRANSKY E, 2009	Multicenter analysis of glucocerebrosidase mutations in Parkinson's disease.	Data collected from 16 centers demonstrate that there is a strong association between *GBA* mutations and Parkinson's disease.	Sidransky et al., 2009
Cluster #1 (Gaucher disease)	HANNUN YA, 2008	Principles of bioactive lipid signaling: lessons from sphingolipids.	The cellular actions of ceramide and other bioactive sphingolipids appear to be crucial for angiogenesis, immune responses, diabetes, aging, cancer biology and degenerative diseases.	Hannun and Obeid, 2008
Cluster #2 (Replacement therapy)	BARTON NW, 1991	Replacement therapy for inherited enzyme deficiency-macrophage-targeted glucocerebrosidase for Gaucher's disease.	Intravenous administration of macrophage-targeted human placental glucocerebrosidase produces objective clinical improvement in patients with type 1 Gaucher disease.	Barton et al., 1991
Cluster #3 (Human spleen)	PENTCHEVPG, 1973	Isolation and characterization of glucocerebrosidase from human placental tissue.	Human placental glucocerebrosidase was purified and characterized as a tetramer composed of 4 catalitically active units whose mass is 60kDa each. The enzyme is most active with glucocerebroside.	Pentchev et al., 1973
Cluster #4 (Molecular analysis)	BEUTLER E, 1995	Gaucher disease. In: The Metabolic and Molecular Bases of Inherited Disease, 7th edition.	This chapter provides a comprehensive coverage of the genetic, molecular, metabolic and clinical underpinnings of Gaucher disease.	Beutler and Grabowski, 1995
Cluster #5 (Kupffer cells)	BRADY RO, 1983	Glucosyl Ceramide Lipidosis: Gaucher's Disease. In: The Metabolic Basis of Inherited Disease, 5th edition.	This chapter reviews the clinical manifestations, pathophysiology, metabolic abnormality and molecular basis of Gaucher disease.	Brady and Barranger, 1983
Cluster #6(Human glucocerebrosidase)	BARTON NW, 1990	Therapeutic response to intravenous infusions of glucocerebrosidase in a patient with Gaucher disease.	This study report the clinical improvement in a child with type 1 Gaucher disease after weekly intravenous infusions of human placental glucocerebrosidase.	Barton et al., 1990
Cluster #7(Sphingolipid precursors)	JECKEL D, 1992	Glucosylceramide is synthesized at the cytosolic surface of various Golgi subfractions.	A truncated ceramide analog was used to show that glucocerebroside is synthetized at the cytosolic surface of the Golgi. Glucosylceramide synthase activity is found in fractions containing marker enzymes for the proximal Golgi, and also in fractions containing distal Golgi markers.	Jeckel et al., 1992
Cluster #8(Pharmacological chaperone)	STEET RA, 2006	The iminosugar isofagomine increases the activity of N370S mutant acid beta-glucosidase in Gaucher fibroblasts by several mechanisms.	The iminosugar isofagomine increases the activity of N370S mutant glucocerebrosidase in Gaucher fibroblasts. A major effect of isofagomine is to act as a pharmacological chaperones to assist the folding and transport of glucocerebrosidase in the endoplasmic reticulum, thereby increasing the lysosomal pool of the enzyme.	Steet et al., 2006
Cluster #9(Glucosylceramide beta-glucosidase)	GLEW RH, 1988	Mammalian glucocerebrosidase: implications for Gaucher's disease.	This review synthetizes information about the molecular biology, chemistry and enzymatic properties of glucocerebrosidase and its relevance to Gaucher disease.	Glew et al., 1988

Taking into account the 10 most cited publications of each cluster, we propose a brief description of the intellectual content of the ten major clusters ranked by their size as follow:

**Cluster #0 (Parkinson's disease)**: This cluster includes publications exploring the links between glucocerebrosidase, glucocerebrosides, and synucleinopathies such as Parkinson's disease and Dementia with Lewy bodies. These focus for example on *GBA* mutations, their impact on Parkinson's disease clinical phenotype, glucocerebrosidase enzymatic activity in Parkinson's disease and possible mechanistic links between glucocerebrosidase deficiency and alpha-synuclein accumulation.

**Cluster #1 (Gaucher disease)**: Publications of this cluster outline the interest of glucocerebrosides as biomarkers for multidrug resistance in cancer cells and explore how the modulation of glucocerebroside metabolism (i.e., the modulation of glucocerebrosidase or glucosylceramide synthase enzymatic activities) can confer or reverse drug resistance in cancer cells.

**Cluster #2 (Replacement therapy)**: This cluster relates to the therapeutic goals and strategies for Gaucher disease. It includes publications evaluating the clinical response to the treatments approved in the treatment of Gaucher disease and relying on enzyme replacement therapy (i.e., use of imiglucerase and previously aglucerase) or substrate reduction therapy (e.g., use of miglustat and eliglustat).

**Cluster #3 (Human spleen)**: This cluster contains publications on the isolation, characterization and comparison of wild-type and mutant glucocerebrosidase from human tissues and cells (i.e., spleen, placenta, fibroblasts). Several publications also concern anionic phospholipids and the activator protein saposin C that promote glucocerebrosidase enzymatic function and contribute to Gaucher disease heterogeneity.

**Cluster #4 (Molecular analysis)**: This cluster englobes publications on the *GBA* gene encoding the lysosomal glucocerebrosidase and its highly homologous pseudogene. Publications can focus on their structure, evolution, heterogeneity and to their mutation and their association with the diagnosis and clinical severity in Gaucher disease.

**Cluster #5 (Kupffer cell):** This cluster contains publications on the cloning and nucleotidic sequence of the *GBA* gene and on the polypeptide sequence of the lysosomal glucocerebrosidase. It contains articles describing the purification of human glucocerebrosidase or its synthesis from cDNA clones for enzyme replacement therapy in Gaucher disease.

**Cluster #6 (Human glucocerebrosidase)**: This cluster is on the correction of glucocerebrosidase deficiency and its therapeutic response. Studies can rely on various strategies including intravenous infusion of glucocerebrosidase (e.g., replacement therapy), bone marrow transplantation in Humans as well as evaluation of gene therapy (i.e., retroviral-mediated gene transfer into bone marrow or hematopoietic cells) in experimental models or patients-derived cells.

**Cluster #7 (Sphingolipid precursors):** This cluster is on the different steps involved in the synthesis of glucocerebrosides and glycosphingolipids, their cellular localization as well as their regulatory function in cellular development and differentiation.

**Cluster #8 (Pharmacological chaperone):** Most publications of this cluster explore the ability of pharmacological chaperone to increase the enzymatic activity of the glucocerebrosidase possibly by decreasing its retention in the endoplasmic reticulum and restoring its trafficking toward the Golgi apparatus and the lysosome.

**Cluster #9 (Glucosylceramide beta-glucosidase)**: This cluster is on the glucocerebrosidase and its mechanisms of activation. Many publications focus on the four saposins (A to D) that are activators of the glucocerebrosidase and especially on saposin C that mutation results in a Gaucher disease-like phenotype in Human.

### Timeline View of the Glucocerebrosides Co-citation Network

**Figure 5:**
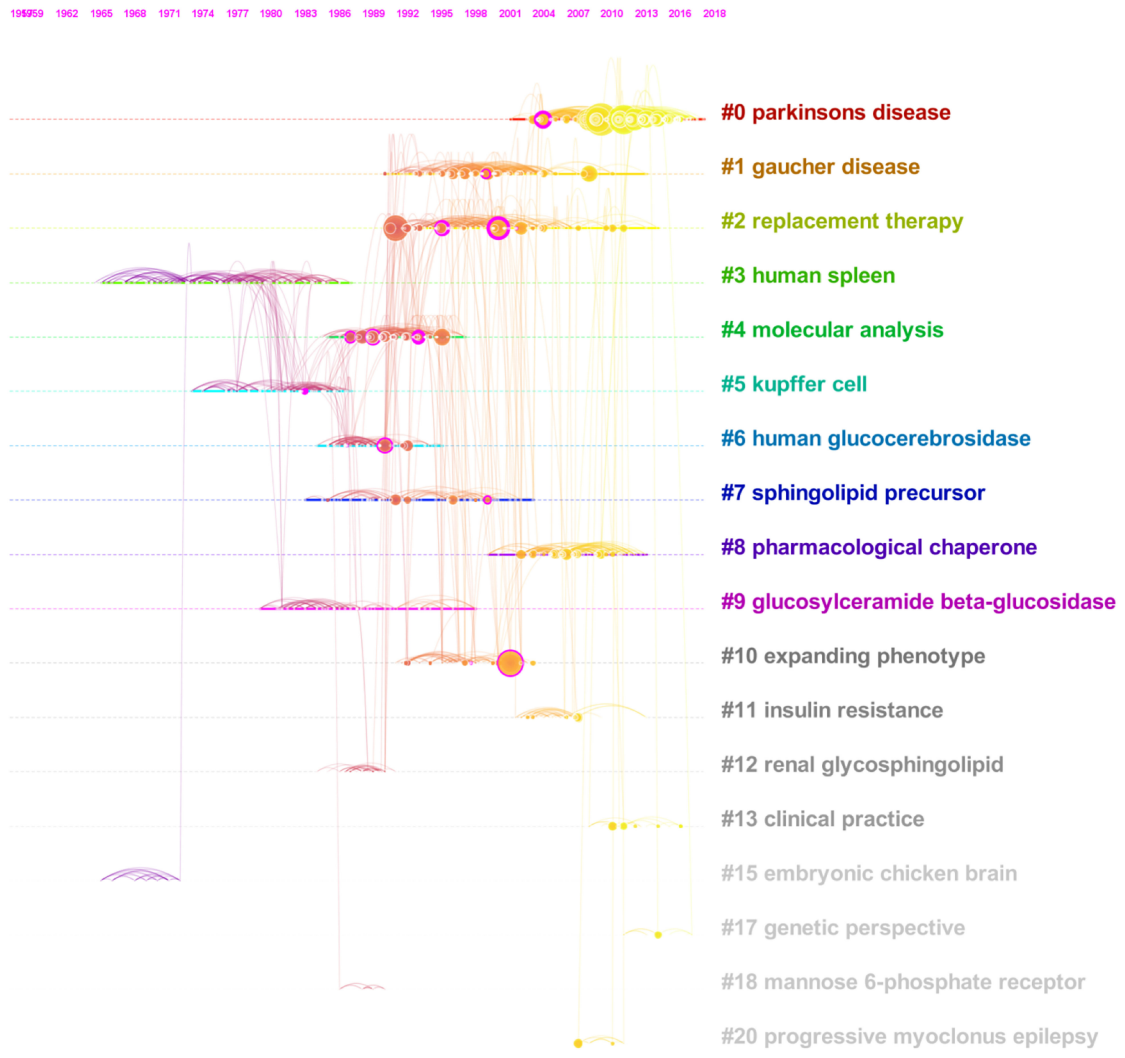
Timeline view of the co-citation network on glucocerebrosides research (1956–2019). The publication dates of articles are placed at the top of the view. Major clusters are labeled on the right.

Clusters were next represented in a timeline view so that nodes of a cluster share a horizontal line. Nodes with a betweenness centrality >0.1 are still represented with a purple ring. This representation helps to understand the temporal characteristics of each cluster and thus the changes in glucocerebrosides research trends over time. As seen in Figure 5, the development of cluster #3 (human spleen; that several publications contributed to the isolation, characterization and comparison of wild-type and mutant glucocerebrosidase from Human tissues and cells) occurred first. Its development was paralleled with that of the smaller cluster #15 (embryonic chicken brain) that largely investigated the composition of the glycolipid fraction of the embryonic chicken brain. Cluster #5 (kupffer cells) developed in the 1970s, reflecting the advances in the sequencing, purification and synthesis of the glucocerebrosidase. Subsequently, the development of the clusters #9 (glucosylceramide beta-glucosidase), #7 (sphingolipid precursor), and then #6 (human glucocerebrosidase) reveals the progress in our understanding on the synthesis and degradation of glucocerebrosides and glycosphingolipids and on the therapeutic response following the correction of glucocerebrosidase deficiency. Cluster #4 (molecular analysis) developed from the 1990, a period of intensive development of tools for genomic analysis 5 years after the Human Genome Project was conceived in 1985. Its publications largely contributed to our knowledge on the structure and variants of the *GBA* gene and its pseudogene and well as their association with phenotypes of Gaucher disease. Cluster #1 (Gaucher disease; that publications evaluate the interest of glucocerebrosides as biomarkers for multidrug resistance in cancer cells) developed in the 1990s. Cluster #2 (replacement therapy) and later cluster #8 (pharmacological chaperone) developed subsequently and helped to define the therapeutic goals and strategies for Gaucher disease. The period of development of these Clusters #2 #8 preceded the U.S. Food and Drug Administration approval of the first effective treatments for Gaucher disease (enzyme replacement therapies; alglucerase in 1991, imiglucerase in 1994) and of substrate reduction therapies (miglustat in 2003 and eliglustat in 2014). Finally, the timeline view evidences that cluster #0 on Parkinson's disease is the most recently formed cluster (Figure 5).

### Emerging Trends in Glucocerebrosides Research

In an attempt to characterize the most recent trends in glucocerebrosides research, we searched for the ten publications with the strongest citation bursts starting after 2016. The number of citations of these articles had a surge during the last four years, suggesting a recent and rapid acceptance and dissemination. All of these top ten publications belong to Cluster #0 on Parkinson's disease, focusing for example on the relationship between *GBA* mutations or polymorphism and the onset, progression and clinical features of Parkinson's disease (Seto-Salvia et al., 2012; Beavan et al., 2015; Brockmann et al., 2015; Gan-Or et al., 2015; Cilia et al., 2016) and on the decrease of glucocerebrosidase activity in blood samples from patients with Parkinson's disease (Alcalay et al., 2015). The title, cluster, burstness score and main conclusion of each of these publications are summarized in Table 5.

**Table 5:** Top 10 publications with subsequent citation bursts starting after 2016 in descending order of burst strength.

**Node's name**	**Title**	**Cluster**	**Burstness (years)**	**Main conclusion**	**References**
ALCALAY RN, 2015	Glucocerebrosidase activity in Parkinson's disease with and without GBA mutations	0	33.75 (2016-2019)	Glucocerebrosidase activity measured in dried blood spots is decreased both in Parkinson's disease patients that are *GBA* mutation carriers and without *GBA* mutations, compared to control individuals.	Alcalay et al., 2015
SCHAPIRA AHV, 2015	Glucocerebrosidase and Parkinson disease: Recent advances	0	26.75 (2016-2019)	This article reviews the pathogenic relationship between glucocerebrosidase deficits and alpha-synuclein pathology and suggests target pathway for the development of therapies against Parkinson's disease.	Schapira, 2015
GAN-OR Z, 2015	Differential effects of severe vs mild GBA mutations on Parkinson disease	0	26.22 (2016-2019)	Carriers of severe *GBA* mutations (those that otherwise cause type 2 and 3 Gaucher disease) have 3 to 4-fold higher risk to develop Parkinson's disease and about 5 years younger age at onset than individuals with mild *GBA* mutations (that cause type 1 Gaucher disease).	Gan-Or et al., 2015
CILIA R, 2016	Survival and dementia in GBA-associated Parkinson's disease: the mutation matters	0	25.86 (2017-2019)	Carriers of severe mutations (that cause type 2 and 3 Gaucher disease) have greater risk for dementia compared to carriers of mild mutations (those that cause type 1 Gaucher disease), but similar mortality risk.	Cilia et al., 2016
BEAVAN M, 2015	Evolution of prodromal clinical markers of Parkinson disease in a GBA mutation-positive cohort	0	23.53 (2016-2019)	This study shows that, as a group, *GBA* mutation positive individuals show clinical features associated with pre-motor and motor features of Parkinson's disease. Among all investigated clinical markers, hyposmia was the most sensitive marker.	Beavan et al., 2015
LIU GQ, 2016	Specifically neuropathic Gaucher's mutations accelerate cognitive decline in Parkinson's	0	21.74 (2017-2019)	Patients with neuropathic *GBA* mutations (cause type 2 and 3 Gaucher disease) have a much more rapid rate of decline in Mini Mental State Examination score that patients with non-neuropathic *GBA* mutations.	Liu et al., 2016
ALCALAY RN, 2014, JAMA NEUROL, V71, P752, DOI	Comparison of Parkinson Risk in Ashkenazi Jewish patients with Gaucher disease and GBA heterozygotes	0	18.79 (2017-2019)	Patients with Gaucher disease and individuals with a mutated allele of *GBA* have an increased age-specific risk for Parkinson's disease compared with control individuals, with a similar magnitude of Parkinson's disease risk by 80 years of age.	Alcalay et al., 2014
BROCKMANN K, 2015	GBA-associated Parkinson's disease: reduced survival and more rapid progression in a prospective longitudinal study	0	17.61 (2017-2019)	The mutational *GBA* status is an important predictor for Parkinson's disease progression. Patients identified as carriers of *GBA* mutation, although younger and with an earlier age at onset, present more rapid progression of motor and cognitive impairments and reduced survival rates.	Brockmann et al., 2015
MAZZULLI JR, 2016	Activation of β-glucocerebrosidase reduces pathological α-synuclein and restores lysosomal function in Parkinson's patient midbrain neurons	0	17.61 (2017-2019)	Small molecule-mediated lysosomal glucocerebrosidase activation could lowered alpha-synuclein levels in induced pluripotent stem cell-derived human midbrain dopaminergic neurons from patients with different Parkinson's disease-linked mutations	Mazzulli et al., 2016
SETO-SALVIA N, 2012, MOVEMENT DISORD, V27, P393, DOI	Glucocerebrosidase mutations confer a greater risk of dementia during parkinson's disease course	0	17.22 (2017-2019)	The individual risk of dementia in Parkinson's disease patients could be increased 6-fold in *GBA* mutation carrier patients compared to noncarrier patients.	Seto-Salvia et al., 2012

## Discussion

This study analyzed the structure, evolution and trends of glucocerebroside research. The first step involved to systematically collect published material relating to glucocerebrosides using the Science Citation Index-Expanded (SCI-E) of the Web of Science Core Collection online database, a major bibliometric database where data about each publication are entered in a uniform structured way (author, title, date, journal name, abstract…). Although as all databases it does not include all articles and in particular miss those published before 1956, it is thus considered as a reliable database enabling an accurate retrieval in title, abstract or keywords searching. Our search formula allowed us to retrieve 5,324 elements published between 1956 and 2019 and referring for example to glucocerebrosides, glucocerebrosidase or glucosylceramide synthase in their title, abstract, author Keywords or Keywords Plus. This dataset was analyzed using CiteSpace, a program adapted to examine the scientific literature in the field of biology and Human health (Chen S. et al., 2019; Chen X. et al., 2019; Huang et al., 2019). Here, it enabled to analyze and present our dataset in a relatively structured, objective and comprehensive way. As such our study can help the researcher to capture the major areas of research relating to glucocerebroside metabolism and their evolution over time.

The 5,324 publications of the dataset were in majority research publications (75.11%) and reviews (10.57%) and were mostly associated to the categories “Biochemistry & Molecular Biology,” “Genetics & Heredity or Medicine,” and “Research & Experimental.” However, the recent burst of the categories “Clinical Neurology” (4th in term of citations; starting in 2012) and “Neuroscience” (7th in term of citations; starting in 2015) as well as the recent burst of keywords tied to neurodegeneration (e.g., “Parkinson's disease” starting in 2014 and “alpha-synuclein” starting in 2013) unequivocally show that the interest for glucocerebrosides in the nervous system increased over the last decade. The different lines of research were also apprehended at the publication level through the co-citation networks analysis. Six of the ten publications with the most co-citation times refer directly to Gaucher disease either on its physiopathology and phenotype (Beutler and Grabowski, 1995, 2001; Grabowski, 2008), its treatment by enzyme replacement therapy (Barton et al., 1991) or substrate reduction (Cox et al., 2000) or its links with the mutation and polymorphism spectrum in the *GBA* gene (Hruska et al., 2008). The four other most-cited publications were published in or after 2009 and refer to Parkinson's disease. These include articles on glucocerebrosidase mutations and Parkinson's disease (Neumann et al., 2009; Sidransky et al., 2009), glucocerebrosidase deficiency in Parkinson's disease (Gegg et al., 2012) or on the mechanistic link between glucocerebrosidase, glucocerebrosides and alpha-synuclein (Mazzulli et al., 2011). The relationship between Gaucher disease and Parkinson's disease has been considered after reports of parkinsonian manifestations among some patients with type 1 Gaucher disease and their first degree relatives (Tayebi et al., 2003; Goker-Alpan et al., 2004; Cherin et al., 2010). Persistent investment in this research has led, among other discoveries, to the finding that ~10 percent of people with Parkinson's have a mutation in one copy of the *GBA* gene (the causing gene of Gaucher disease) (Sidransky et al., 2009) that is study with the most citations and also the highest citation bursts across our entire dataset. On the other hand, it is now proposed that neurologic manifestations in Gaucher disease could rather represent a phenotypic continuum, ranging from hydrops fetalis, at the severe end of type 2 Gaucher disease, to extrapyramidal syndrome (e.g., parkinsonism) at the mild end in type 1 Gaucher disease (Stirnemann et al., 2017).

The clustering analysis of the co-citation network allowed us to get a more specific view on the major areas of research relating to glucocerebrosides. Beside a major cluster on Parkinson's disease (cluster #0), we identified specific clusters centered on the *GBA* gene (#4), glucocerebrosidase protein (#5), mutant glucocerebrosidase in Gaucher disease (#3), glucocerebrosidase activation (#9), therapeutic effects of the correction of glucocerebrosidase activity (#6), pharmacological chaperones aimed to increase glucocerebrosidase activity (#8), or on sphingolipid metabolism (#7). In Supplementary Table 1 and in the result section, we provide for each of these clusters the 10 most cited publications and propose a description of their intellectual content so that the reader can efficiently apprehend the bases of these lines of research. Many of these clusters' publications published before the surge of glucocerebroside-related research in Parkinson's disease are highly relevant for this disease. It has to be noted, for example, that (i) the *GBA* mutations considered as severe in Gaucher disease (those that cause type 2 and 3 Gaucher disease) are also associated with a 3 to 4 fold higher risk to develop Parkinson's disease compared to mild *GBA* mutation (that cause type 1 Gaucher disease) (Gan-Or et al., 2015); (ii) glucocerebrosidase mutation and deficiency that is associated to Gaucher disease could otherwise contribute to alterations in alpha-synuclein homeostasis, lysosomal chaperone-mediated autophagy and immunity pathways involved in Parkinson's disease pathogenesis (Murphy et al., 2014; Kitatani et al., 2015; Aflaki et al., 2016; Pandey et al., 2017); and (iii) therapeutic strategies that were first considered in Gaucher disease are now tested in clinical studies as potential disease-modifying therapeutics against Parkinson's disease (Peterschmitt et al., 2019; Riboldi and Di Fonzo, 2019). Thus, our study may help the reader to capture publications and intellectual bases of the major areas of research relating to glucocerebrosides and glucocerebrosidase with relevance not only to Gaucher disease but also to other human diseases including synucleinopathies.

The clustering analysis of the co-citation network also revealed an important cluster of glucocerebrosides research on multidrug resistance (cluster #1). Most-cited articles of this cluster support the notion that cellular drug resistance phenomena in cancer are aligned to alterations in glucosylceramide metabolism (Lavie et al., 1996) and points to the effects of glucosylceramide synthase in protecting tumor cells from chemotherapy (Lavie et al., 1997; Liu et al., 1999; Ogretmen and Hannun, 2004). The connections between the roles of glucosylceramides in cancer pathogenesis on one hand and Gaucher disease and Parkinson's disease on the other hand are rarely evoked. Thus, our network clustering analysis suggest that more exchange between these research fields could improve our understanding of the biological processes involved in these pathologies. This view is particularly relevant as epidemiology studies show that a history of several cancers is less likely among patients with Dementia with Lewy bodies or Parkinson's disease (Bajaj et al., 2010; Boot et al., 2013). Importantly, the molecular pathways involved in tumorigenesis (e.g., cell proliferation) are intertwined with those involved in neurodegeneration (e.g., cell death) (Houck et al., 2018; Seo and Park, 2019). For instance, loss of proteostasis, deregulated nutrient sensing, mitochondrial dysfunction, oxidative imbalance and altered intercellular communication are biological pathways involved in both cancer and neurodegeneration and are all regulated by glycosphingolipids. Thus, our results pinpoint the need for more exchanges between the scientific communities studying glycosphingolipids—and more broadly lipid-related risk factors and mechanisms—in cancer, lysosomal storage disorders, and neurodegenerative diseases. As such, sharing database, cross-checking data or confronting expert opinions on glucocerebrosides at the crossroad of these pathologies could facilitate advances in these fields.

In an attempt to catch the most recent trends in glucocerebrosides research, we searched for the ten publications in our dataset with the strongest citation bursts starting after 2016. This analysis confirmed that Parkinson's disease remains to date the most active area of research on glucocerebrosides as all the selected publications were part of the cluster #0. These publications relate to the glucocerebrosidase enzymatic activity in dried blood spots from patients (Alcalay et al., 2015), to the influence of *GBA* mutation on the development of cognitive impairment, dementia, and death in Parkinson's disease patients (Seto-Salvia et al., 2012; Alcalay et al., 2014; Beavan et al., 2015; Brockmann et al., 2015; Gan-Or et al., 2015; Cilia et al., 2016; Liu et al., 2016) and to the pathogenic links between glucocerebrosidase, glucocerebrosides, and alpha-synuclein and the therapeutic potential of glucocerebrosidase-enhancing molecules (Schapira, 2015; Mazzulli et al., 2016). These studies thus showed a good acceptance among the scientific community and will likely made a significant contribution to the knowledge structure of glucocerebrosides research in the future.

In conclusion, our work provides a reference study to apprehend glucocerebrosides-related literature at a global level. It delineates the major research areas and diseases around which it developed from 1956 to present (i.e., the therapeutic goals and strategies for Gaucher disease, drug resistance in cancer, *GBA* gene mutation and polymorphisms analysis, characterization, and comparison of wild-type and mutant glucocerebrosidase, glycosphingolipids metabolism) and undoubtedly identify Parkinson's disease and synucleinopathies as the most active area of research on glucocerebrosides at the present time. Our study can help the researcher to capture the intellectual bases of glucocerebrosides research and to enhance transdisciplinary research among the different scientific communities involved in glucocerebrosides research.

## Data Availability Statement

Publicly available datasets were analyzed in this study. This data can be found here: Science Citation Index-Expanded (SCI-E) of the Web of Science Core Collection online database (Thomson Reuters) hosted by Clarivate Analytics.

## Author Contributions

MD and M-AB performed the literature search and analysis. BG and DD reviewed and edited the manuscript. M-CC-H contributed to the structure of the manuscript, reviewed and edited it. KB contributed to the conceptualization, methodology and execution of the study and wrote the original draft of the manuscript. All authors contributed to the article and approved the submitted version.

## Conflict of Interest

The authors declare that the research was conducted in the absence of any commercial or financial relationships that could be construed as a potential conflict of interest.
