# Supplementary material for: Trends in Glucocerebrosides Research: A Systematic Review
Source: Front Physiol. 2020 Oct 29;11:558090. doi: 10.3389/fphys.2020.558090 (PMC7658098; doi:10.3389/fphys.2020.558090)
Supplement: Supplementary file 1 [file Table_1.DOCX]

**Supplemental table 1.** **Most frequently cited references in the ten largest document co-citation clusters ranked by citation counts.** Citations is the number of citation of the publication in the network. Burst quantifies the surge of the citation for a specific period of time. Betweenness centrality quantifies the importance of the publication’s position in the cluster. Sigma combines betweenness centrality and burst rate to provide an indication of the transformative strength of the publication in the network.

| **Cluster ID and label** | **Citations** | **Burst** | **Centrality** | **Sigma** | **Cited reference** |
| --- | --- | --- | --- | --- | --- |
| Cluster #0 (Parkinson's disease) | 378 | 138.24 | 0.05 | 1316.60 | (1) |
|  | 287 | 105.63 | 0.01 | 1.93 | (2) |
|  | 192 | 69.14 | 0.04 | 18.34 | (3) |
|  | 166 | 67.26 | 0.01 | 1.55 | (4) |
|  | 156 | 57.93 | 0.00 | 1.00 | (5) |
|  | 111 | 40.26 | 0.01 | 1.57 | (6) |
|  | 109 | 48.76 | 0.00 | 1.22 | (7) |
|  | 107 | 41.09 | 0.03 | 3.22 | (8) |
|  | 104 | 41.92 | 0.00 | 1.21 | (9) |
|  | 102 | 37.71 | 0.01 | 1.54 | (10) |
| Cluster #1 (Gaucher disease) | 100 | 36.97 | 0.00 | 1.13 | (11) |
|  | 84 | 40.75 | 0.01 | 1.31 | (12) |
|  | 73 | 35.56 | 0.08 | 15.28 | (13) |
|  | 69 | 35.67 | 0.02 | 1.89 | (14) |
|  | 64 | 30.58 | 0.14 | 62.55 | (15) |
|  | 56 | 26.36 | 0.02 | 1.56 | (16) |
|  | 55 | 26.62 | 0.05 | 4.14 | (17) |
|  | 51 | 26.32 | 0.02 | 1.63 | (18) |
|  | 49 | 25.95 | 0.03 | 2.16 | (19) |
|  | 48 | 24.32 | 0.08 | 6.23 | (20) |
| Cluster #2 (Replacement therapy) | 168 | 79.65 | 0.02 | 4.09 | (21) |
|  | 115 | 52.32 | 0.27 | 219699.69 | (22) |
|  | 82 | 39.02 | 0.08 | 22.94 | (23) |
|  | 66 | 32.64 | 0.01 | 1.58 | (24) |
|  | 65 | 31.33 | 0.39 | 32501.36 | (25) |
|  | 63 | 30.36 | 0.01 | 1.26 | (26) |
|  | 60 | 26.45 | 0.03 | 2.05 | (27) |
|  | 53 | 26.5 | 0.01 | 1.22 | (28) |
|  | 51 | 24.44 | 0.01 | 1.32 | (29) |
|  | 50 | 25.9 | 0.00 | 1.06 | (30) |
| Cluster #3 (Human spleen) | 18 | 12.27 | 0.06 | 2.12 | (31) |
|  | 18 | 11.67 | 0.00 | 1.01 | (32) |
|  | 14 | 9.26 | 0.02 | 1.23 | (33) |
|  | 12 | 8.11 | 0.07 | 1.76 | (34) |
|  | 12 | 7.99 | 0.01 | 1.11 | (35) |
|  | 12 | 8.19 | 0.01 | 1.07 | (36) |
|  | 12 | 8.2 | 0.00 | 1.03 | (37) |
|  | 11 | 7.08 | 0.00 | 1.01 | (38) |
|  | 10 | 6.84 | 0.01 | 1.09 | (39) |
|  | 10 | 6.7 | 0.01 | 1.04 | (40) |
| Cluster #4 (Molecular analysis) | 124 | 59.72 | 0.06 | 27.09 | (41) |
|  | 102 | 48.9 | 0.15 | 891.75 | (42) |
|  | 86 | 45.37 | 0.17 | 1041.83 | (43) |
|  | 85 | 40.79 | 0.01 | 1.48 | (44) |
|  | 80 | 40.56 | 0.01 | 1.77 | (45) |
|  | 79 | 41.01 | 0.01 | 1.29 | (46) |
|  | 61 | 28.62 | 0.01 | 1.35 | (47) |
|  | 60 | 30.02 | 0.02 | 1.93 | (48) |
|  | 58 | 27.2 | 0.02 | 1.73 | (49) |
|  | 55 | 28.63 | 0.01 | 1.25 | (50) |
| Cluster #5 (Kupffer cells) | 41 | 25.37 | 0.37 | 2974.81 | (51) |
|  | 37 | 21.47 | 0.01 | 1.35 | (52) |
|  | 36 | 23.17 | 0.06 | 4.28 | (53) |
|  | 34 | 22.88 | 0.10 | 7.98 | (54) |
|  | 30 | 17.4 | 0.00 | 1.03 | (55) |
|  | 21 | 12 | 0.00 | 1.03 | (56) |
|  | 19 | 12.21 | 0.02 | 1.26 | (57) |
|  | 18 | 11.34 | 0.02 | 1.30 | (58) |
|  | 17 | 11.32 | 0.02 | 1.27 | (59) |
|  | 17 | 9.91 | 0.00 | 1.04 | (60) |
| Cluster #6  (Human glucocerebrosidase) | 79 | 35.81 | 0.16 | 220.66 | (61) |
|  | 58 | 30.06 | 0.01 | 1.22 | (62) |
|  | 36 | 20.55 | 0.01 | 1.34 | (63) |
|  | 23 | 12.7 | 0.01 | 1.19 | (64) |
|  | 22 | 13.3 | 0.00 | 1.00 | (65) |
|  | 21 | 12.2 | 0.01 | 1.08 | (66) |
|  | 16 | 8.83 | 0.02 | 1.23 | (67) |
|  | 15 | 8.91 | 0.03 | 1.33 | (68) |
|  | 14 | 7.06 | 0.03 | 1.21 | (69) |
|  | 14 | 8.89 | 0.00 | 1.01 | (70) |
| Cluster #7  (Sphingolipid precursors) | 57 | 26.53 | 0.02 | 1.57 | (71) |
|  | 55 | 27.15 | 0.01 | 1.44 | (72) |
|  | 45 | 24.28 | 0.02 | 1.67 | (73) |
|  | 41 | 22 | 0.11 | 10.59 | (74) |
|  | 38 | 17.79 | 0.06 | 2.66 | (75) |
|  | 35 | 18.96 | 0.01 | 1.20 | (76) |
|  | 23 | 14.05 | 0.01 | 1.20 | (77) |
|  | 21 | 11.84 | 0.06 | 2.06 | (78) |
|  | 20 | 11.54 | 0.01 | 1.16 | (79) |
|  | 19 | 11.52 | 0.00 | 1.00 | (80) |
| Cluster #8  (Pharmacological chaperone) | 73 | 30.24 | 0.03 | 2.43 | (81) |
|  | 64 | 32.03 | 0.07 | 8.30 | (82) |
|  | 61 | 29.69 | 0.00 | 1.09 | (83) |
|  | 56 | 26.94 | 0.03 | 2.46 | (84) |
|  | 51 | 19.69 | 0.04 | 2.25 | (85) |
|  | 44 | 18.76 | 0.01 | 1.23 | (86) |
|  | 43 | 22.79 | 0.01 | 1.16 | (87) |
|  | 41 | 18.65 | 0.00 | 1.06 | (88) |
|  | 40 | 21.08 | 0.02 | 1.62 | (89) |
|  | 36 | 16.15 | 0.01 | 1.12 | (90) |
| Cluster #9  (Glucosylceramide beta-glucosidase) | 23 | 13.67 | 0.00 | 1.02 | (91) |
|  | 21 | 12.48 | 0.06 | 2.03 | (92) |
|  | 15 | 8.28 | 0.01 | 1.04 | (93) |
|  | 14 | 8.31 | 0.00 | 1.02 | (94) |
|  | 12 | 5.9 | 0.06 | 1.38 | (95) |
|  | 10 | 6.17 | 0.02 | 1.16 | (96) |
|  | 10 | 6.16 | 0.00 | 1.00 | (97) |
|  | 8 | 5.29 | 0.03 | 1.16 | (98) |
|  | 8 | 5.13 | 0.03 | 1.14 | (99) |
|  | 7 | 4.7 | 0.02 | 1.07 | (100) |

1. Sidransky E, Nalls MA, Aasly JO, Aharon-Peretz J, Annesi G, Barbosa ER, et al. Multicenter analysis of glucocerebrosidase mutations in Parkinson's disease. *N Engl J Med* (2009) 361(17):1651-61. Epub 2009/10/23. doi: 361/17/1651 [pii]

10.1056/NEJMoa0901281. PubMed PMID: 19846850; PubMed Central PMCID: PMC2856322.

2. Mazzulli JR, Xu YH, Sun Y, Knight AL, McLean PJ, Caldwell GA, et al. Gaucher disease glucocerebrosidase and alpha-synuclein form a bidirectional pathogenic loop in synucleinopathies. *Cell* (2011) 146(1):37-52. Epub 2011/06/28. doi: S0092-8674(11)00601-5 [pii]

10.1016/j.cell.2011.06.001. PubMed PMID: 21700325; PubMed Central PMCID: PMC3132082.

3. Neumann J, Bras J, Deas E, O'Sullivan SS, Parkkinen L, Lachmann RH, et al. Glucocerebrosidase mutations in clinical and pathologically proven Parkinson's disease. *Brain* (2009) 132(Pt 7):1783-94. Epub 2009/03/17. doi: awp044 [pii]

10.1093/brain/awp044. PubMed PMID: 19286695; PubMed Central PMCID: PMC2702833.

4. Gegg ME, Burke D, Heales SJ, Cooper JM, Hardy J, Wood NW, et al. Glucocerebrosidase deficiency in substantia nigra of parkinson disease brains. *Ann Neurol* (2012) 72(3):455-63. Epub 2012/10/05. doi: 10.1002/ana.23614. PubMed PMID: 23034917.

5. Hruska KS, LaMarca ME, Scott CR, Sidransky E. Gaucher disease: mutation and polymorphism spectrum in the glucocerebrosidase gene (GBA). *Hum Mutat* (2008) 29(5):567-83. Epub 2008/03/14. doi: 10.1002/humu.20676. PubMed PMID: 18338393.

6. Sardi SP, Clarke J, Kinnecom C, Tamsett TJ, Li L, Stanek LM, et al. CNS expression of glucocerebrosidase corrects alpha-synuclein pathology and memory in a mouse model of Gaucher-related synucleinopathy. *Proc Natl Acad Sci U S A* (2011) 108(29):12101-6. Epub 2011/07/07. doi: 1108197108 [pii]

10.1073/pnas.1108197108. PubMed PMID: 21730160; PubMed Central PMCID: PMC3141921.

7. Nalls MA, Duran R, Lopez G, Kurzawa-Akanbi M, McKeith IG, Chinnery PF, et al. A multicenter study of glucocerebrosidase mutations in dementia with Lewy bodies. *JAMA Neurol* (2013) 70(6):727-35. Epub 2013/04/17. doi: 10.1001/jamaneurol.2013.1925. PubMed PMID: 23588557; PubMed Central PMCID: PMCPMC3841974.

8. Cullen V, Sardi SP, Ng J, Xu YH, Sun Y, Tomlinson JJ, et al. Acid beta-glucosidase mutants linked to Gaucher disease, Parkinson disease, and Lewy body dementia alter alpha-synuclein processing. *Ann Neurol* (2011) 69(6):940-53. Epub 2011/04/08. doi: 10.1002/ana.22400. PubMed PMID: 21472771.

9. Sidransky E, Lopez G. The link between the GBA gene and parkinsonism. *Lancet Neurol* (2012) 11(11):986-98. Epub 2012/10/20. doi: S1474-4422(12)70190-4 [pii]

10.1016/S1474-4422(12)70190-4. PubMed PMID: 23079555.

10. Gan-Or Z, Giladi N, Rozovski U, Shifrin C, Rosner S, Gurevich T, et al. Genotype-phenotype correlations between GBA mutations and Parkinson disease risk and onset. *Neurology* (2008) 70(24):2277-83. Epub 2008/04/25. doi: 10.1212/01.wnl.0000304039.11891.29. PubMed PMID: 18434642.

11. Hannun YA, Obeid LM. Principles of bioactive lipid signalling: lessons from sphingolipids. *Nat Rev Mol Cell Biol* (2008) 9(2):139-50. Epub 2008/01/25. doi: 10.1038/nrm2329. PubMed PMID: 18216770.

12. Liu YY, Han TY, Giuliano AE, Cabot MC. Expression of glucosylceramide synthase, converting ceramide to glucosylceramide, confers adriamycin resistance in human breast cancer cells. *J Biol Chem* (1999) 274(2):1140-6. Epub 1999/01/05. doi: 10.1074/jbc.274.2.1140. PubMed PMID: 9873062.

13. Lavie Y, Cao H, Volner A, Lucci A, Han TY, Geffen V, et al. Agents that reverse multidrug resistance, tamoxifen, verapamil, and cyclosporin A, block glycosphingolipid metabolism by inhibiting ceramide glycosylation in human cancer cells. *J Biol Chem* (1997) 272(3):1682-7. Epub 1997/01/17. doi: 10.1074/jbc.272.3.1682. PubMed PMID: 8999846.

14. Lavie Y, Cao H, Bursten SL, Giuliano AE, Cabot MC. Accumulation of glucosylceramides in multidrug-resistant cancer cells. *J Biol Chem* (1996) 271(32):19530-6. Epub 1996/08/09. doi: 10.1074/jbc.271.32.19530. PubMed PMID: 8702646.

15. Lee L, Abe A, Shayman JA. Improved inhibitors of glucosylceramide synthase. *J Biol Chem* (1999) 274(21):14662-9. Epub 1999/05/18. doi: 10.1074/jbc.274.21.14662. PubMed PMID: 10329660.

16. Ogretmen B, Hannun YA. Biologically active sphingolipids in cancer pathogenesis and treatment. *Nat Rev Cancer* (2004) 4(8):604-16. Epub 2004/08/03. doi: 10.1038/nrc1411. PubMed PMID: 15286740.

17. Lucci A, Cho WI, Han TY, Giuliano AE, Morton DL, Cabot MC. Glucosylceramide: a marker for multiple-drug resistant cancers. *Anticancer Res* (1998) 18(1B):475-80. Epub 1998/05/06. PubMed PMID: 9568165.

18. Hannun YA. Functions of ceramide in coordinating cellular responses to stress. *Science* (1996) 274(5294):1855-9. Epub 1996/12/13. doi: 10.1126/science.274.5294.1855. PubMed PMID: 8943189.

19. Radin NS, Shayman JA, Inokuchi J. Metabolic effects of inhibiting glucosylceramide synthesis with PDMP and other substances. *Adv Lipid Res* (1993) 26:183-213. Epub 1993/01/01. PubMed PMID: 8379450.

20. Abe A, Radin NS, Shayman JA, Wotring LL, Zipkin RE, Sivakumar R, et al. Structural and stereochemical studies of potent inhibitors of glucosylceramide synthase and tumor cell growth. *J Lipid Res* (1995) 36(3):611-21. Epub 1995/03/01. PubMed PMID: 7775872.

21. Barton NW, Brady RO, Dambrosia JM, Di Bisceglie AM, Doppelt SH, Hill SC, et al. Replacement therapy for inherited enzyme deficiency--macrophage-targeted glucocerebrosidase for Gaucher's disease. *N Engl J Med* (1991) 324(21):1464-70. Epub 1991/05/23. doi: 10.1056/NEJM199105233242104. PubMed PMID: 2023606.

22. Cox T, Lachmann R, Hollak C, Aerts J, van Weely S, Hrebicek M, et al. Novel oral treatment of Gaucher's disease with N-butyldeoxynojirimycin (OGT 918) to decrease substrate biosynthesis. *Lancet* (2000) 355(9214):1481-5. Epub 2000/05/09. doi: 10.1016/S0140-6736(00)02161-9. PubMed PMID: 10801168.

23. Weinreb NJ, Charrow J, Andersson HC, Kaplan P, Kolodny EH, Mistry P, et al. Effectiveness of enzyme replacement therapy in 1028 patients with type 1 Gaucher disease after 2 to 5 years of treatment: a report from the Gaucher Registry. *Am J Med* (2002) 113(2):112-9. Epub 2002/07/23. doi: 10.1016/s0002-9343(02)01150-6. PubMed PMID: 12133749.

24. Beutler E, Kay A, Saven A, Garver P, Thurston D, Dawson A, et al. Enzyme replacement therapy for Gaucher disease. *Blood* (1991) 78(5):1183-9. Epub 1991/09/01. PubMed PMID: 1878585.

25. Grabowski GA, Barton NW, Pastores G, Dambrosia JM, Banerjee TK, McKee MA, et al. Enzyme therapy in type 1 Gaucher disease: comparative efficacy of mannose-terminated glucocerebrosidase from natural and recombinant sources. *Ann Intern Med* (1995) 122(1):33-9. Epub 1995/01/01. doi: 10.7326/0003-4819-122-1-199501010-00005. PubMed PMID: 7985893.

26. Rosenthal DI, Doppelt SH, Mankin HJ, Dambrosia JM, Xavier RJ, McKusick KA, et al. Enzyme replacement therapy for Gaucher disease: skeletal responses to macrophage-targeted glucocerebrosidase. *Pediatrics* (1995) 96(4 Pt 1):629-37. Epub 1995/10/01. PubMed PMID: 7567322.

27. Pastores GM, Weinreb NJ, Aerts H, Andria G, Cox TM, Giralt M, et al. Therapeutic goals in the treatment of Gaucher disease. *Semin Hematol* (2004) 41(4 Suppl 5):4-14. Epub 2004/10/07. PubMed PMID: 15468045.

28. Pastores GM, Sibille AR, Grabowski GA. Enzyme therapy in Gaucher disease type 1: dosage efficacy and adverse effects in 33 patients treated for 6 to 24 months. *Blood* (1993) 82(2):408-16. Epub 1993/07/15. PubMed PMID: 8392397.

29. Lukina E, Watman N, Arreguin EA, Banikazemi M, Dragosky M, Iastrebner M, et al. A phase 2 study of eliglustat tartrate (Genz-112638), an oral substrate reduction therapy for Gaucher disease type 1. *Blood* (2010) 116(6):893-9. Epub 2010/05/05. doi: 10.1182/blood-2010-03-273151. PubMed PMID: 20439622; PubMed Central PMCID: PMCPMC2924227.

30. Figueroa ML, Rosenbloom BE, Kay AC, Garver P, Thurston DW, Koziol JA, et al. A less costly regimen of alglucerase to treat Gaucher's disease. *N Engl J Med* (1992) 327(23):1632-6. Epub 1992/12/03. doi: 10.1056/NEJM199212033272304. PubMed PMID: 1435900.

31. Pentchev PG, Brady RO, Hibbert SR, Gal AE, Shapiro D. Isolation and characterization of glucocerebrosidase from human placental tissue. *J Biol Chem* (1973) 248(15):5256-61. Epub 1973/08/10. PubMed PMID: 4768898.

32. Basu A, Glew RH, Daniels LB, Clark LS. Activators of spleen glucocerebrosidase from controls and patients with various forms of Gaucher's disease. *J Biol Chem* (1984) 259(3):1714-9. Epub 1984/02/10. PubMed PMID: 6693432.

33. Glew RH, Daniels LB, Clark LS, Hoyer SW. Enzymic differentiation of neurologic and nonneurologic forms of Gaucher's disease. *J Neuropathol Exp Neurol* (1982) 41(6):630-41. Epub 1982/11/01. doi: 10.1097/00005072-198211000-00006. PubMed PMID: 6813430.

34. Peters SP, Coyle P, Glew RH. Differentiation of beta-glucocerebrosidase from beta-glucosidase in human tissues using sodium taurocholate. *Arch Biochem Biophys* (1976) 175(2):569-82. Epub 1976/08/01. doi: 10.1016/0003-9861(76)90547-6. PubMed PMID: 958319.

35. Pentchev PG, Brady RO, Blair HE, Britton DE, Sorrell SH. Gaucher disease: isolation and comparison of normal and mutant glucocerebrosidase from human spleen tissue. *Proc Natl Acad Sci U S A* (1978) 75(8):3970-3. Epub 1978/08/01. doi: 10.1073/pnas.75.8.3970. PubMed PMID: 29293; PubMed Central PMCID: PMCPMC392911.

36. Ho MW, O'Brien JS. Gaucher's disease: deficiency of 'acid' -glucosidase and reconstitution of enzyme activity in vitro. *Proc Natl Acad Sci U S A* (1971) 68(11):2810-3. Epub 1971/11/01. PubMed PMID: 5288260; PubMed Central PMCID: PMCPMC389531.

37. Brady RO. Glucosyl Ceramide Lipidosis: Gaucher's Disease. In: J. B. Stanbury JBWaDSF, editors, editor. *The Metabolic Basis of Inherited Disease, 4th edition*. 4 ed. New York: McGraw-Hill (1978). p. 731-46.

38. Wenger DA, Roth S. Homozygote and heterozygote identification. *Prog Clin Biol Res* (1982) 95:551-72. Epub 1982/01/01. PubMed PMID: 6812086.

39. Ho MW. Identity of 'acid' beta-glucosidase and glucocerebrosidase in human spleen. *Biochem J* (1973) 136(3):721-9. Epub 1973/11/01. doi: 10.1042/bj1360721. PubMed PMID: 4780697; PubMed Central PMCID: PMCPMC1166008.

40. Mueller OT, Rosenberg A. Activation of membrane-bound glucosylceramide: beta-glucosidase in fibroblasts cultured from normal and glucosylceramidotic human skin. *J Biol Chem* (1979) 254(9):3521-5. Epub 1979/05/10. PubMed PMID: 429368.

41. Beutler E, Grabowski GA. Gaucher disease. In: Scriver CR BA, Sly WS and Valle D, editors, editor. *The Metabolic and Molecular Bases of Inherited Disease*. New York: McGraw-Hill (1995). p. 2641–70.

42. Horowitz M, Wilder S, Horowitz Z, Reiner O, Gelbart T, Beutler E. The human glucocerebrosidase gene and pseudogene: structure and evolution. *Genomics* (1989) 4(1):87-96. Epub 1989/01/01. doi: 10.1016/0888-7543(89)90319-4. PubMed PMID: 2914709.

43. Tsuji S, Choudary PV, Martin BM, Stubblefield BK, Mayor JA, Barranger JA, et al. A mutation in the human glucocerebrosidase gene in neuronopathic Gaucher's disease. *N Engl J Med* (1987) 316(10):570-5. Epub 1987/03/05. doi: 10.1056/NEJM198703053161002. PubMed PMID: 2880291.

44. Zimran A, Sorge J, Gross E, Kubitz M, West C, Beutler E. Prediction of severity of Gaucher's disease by identification of mutations at DNA level. *Lancet* (1989) 2(8659):349-52. Epub 1989/08/12. doi: 10.1016/s0140-6736(89)90536-9. PubMed PMID: 2569551.

45. Tsuji S, Martin BM, Barranger JA, Stubblefield BK, LaMarca ME, Ginns EI. Genetic heterogeneity in type 1 Gaucher disease: multiple genotypes in Ashkenazic and non-Ashkenazic individuals. *Proc Natl Acad Sci U S A* (1988) 85(7):2349-52. Epub 1988/04/01. doi: 10.1073/pnas.85.7.2349. PubMed PMID: 3353383; PubMed Central PMCID: PMCPMC279989.

46. Beutler E. Gaucher disease: new molecular approaches to diagnosis and treatment. *Science* (1992) 256(5058):794-9. Epub 1992/05/08. doi: 10.1126/science.1589760. PubMed PMID: 1589760.

47. Zimran A, Sorge J, Gross E, Kubitz M, West C, Beutler E. A glucocerebrosidase fusion gene in Gaucher disease. Implications for the molecular anatomy, pathogenesis, and diagnosis of this disorder. *J Clin Invest* (1990) 85(1):219-22. Epub 1990/01/01. doi: 10.1172/JCI114415. PubMed PMID: 2295698; PubMed Central PMCID: PMCPMC296408.

48. Zimran A, Kay A, Gelbart T, Garver P, Thurston D, Saven A, et al. Gaucher disease. Clinical, laboratory, radiologic, and genetic features of 53 patients. *Medicine (Baltimore)* (1992) 71(6):337-53. Epub 1992/11/01. PubMed PMID: 1435229.

49. Latham T, Grabowski GA, Theophilus BD, Smith FI. Complex alleles of the acid beta-glucosidase gene in Gaucher disease. *Am J Hum Genet* (1990) 47(1):79-86. Epub 1990/07/01. PubMed PMID: 2349952; PubMed Central PMCID: PMCPMC1683763.

50. Beutler E, Gelbart T, Kuhl W, Sorge J, West C. Identification of the second common Jewish Gaucher disease mutation makes possible population-based screening for the heterozygous state. *Proc Natl Acad Sci U S A* (1991) 88(23):10544-7. Epub 1991/12/01. doi: 10.1073/pnas.88.23.10544. PubMed PMID: 1961718; PubMed Central PMCID: PMCPMC52965.

51. Brady RO, Barranger JA. Glucosyl Ceramide Lipidosis: Gaucher's Disease. In: J. B. Stanbury JBW, D. S. Fredrickson, J. L. Goldstein, M. S. Brown, editors, editor. *The Metabolic Basis of Inherited Disease, 5th edition*. New York: McGraw-Hill (1983). p. 842-56.

52. Sorge J, West C, Westwood B, Beutler E. Molecular cloning and nucleotide sequence of human glucocerebrosidase cDNA. *Proc Natl Acad Sci U S A* (1985) 82(21):7289-93. Epub 1985/11/01. doi: 10.1073/pnas.82.21.7289. PubMed PMID: 3864160; PubMed Central PMCID: PMCPMC390835.

53. Ginns EI, Brady RO, Pirruccello S, Moore C, Sorrell S, Furbish FS, et al. Mutations of glucocerebrosidase: discrimination of neurologic and non-neurologic phenotypes of Gaucher disease. *Proc Natl Acad Sci U S A* (1982) 79(18):5607-10. Epub 1982/09/01. doi: 10.1073/pnas.79.18.5607. PubMed PMID: 6957882; PubMed Central PMCID: PMCPMC346953.

54. Furbish FS, Blair HE, Shiloach J, Pentchev PG, Brady RO. Enzyme replacement therapy in Gaucher's disease: large-scale purification of glucocerebrosidase suitable for human administration. *Proc Natl Acad Sci U S A* (1977) 74(8):3560-3. Epub 1977/08/01. doi: 10.1073/pnas.74.8.3560. PubMed PMID: 269414; PubMed Central PMCID: PMCPMC431631.

55. Tsuji S, Choudary PV, Martin BM, Winfield S, Barranger JA, Ginns EI. Nucleotide sequence of cDNA containing the complete coding sequence for human lysosomal glucocerebrosidase. *J Biol Chem* (1986) 261(1):50-3. Epub 1986/01/05. PubMed PMID: 3001061.

56. Ginns EI, Choudary PV, Tsuji S, Martin B, Stubblefield B, Sawyer J, et al. Gene mapping and leader polypeptide sequence of human glucocerebrosidase: implications for Gaucher disease. *Proc Natl Acad Sci U S A* (1985) 82(20):7101-5. Epub 1985/10/01. doi: 10.1073/pnas.82.20.7101. PubMed PMID: 3863141; PubMed Central PMCID: PMCPMC391318.

57. Ginns EI, Tegelaers FP, Barneveld R, Galjaard H, Reuser AJ, Brady RO, et al. Determination of Gaucher's disease phenotypes with monoclonal antibody. *Clin Chim Acta* (1983) 131(3):283-7. Epub 1983/07/15. doi: 10.1016/0009-8981(83)90097-9. PubMed PMID: 6883722.

58. Erickson AH, Ginns EI, Barranger JA. Biosynthesis of the lysosomal enzyme glucocerebrosidase. *J Biol Chem* (1985) 260(26):14319-24. Epub 1985/11/15. PubMed PMID: 3932353.

59. Ginns EI, Choudary PV, Martin BM, Winfield S, Stubblefield B, Mayor J, et al. Isolation of cDNA clones for human beta-glucocerebrosidase using the lambda gt11 expression system. *Biochem Biophys Res Commun* (1984) 123(2):574-80. Epub 1984/09/17. doi: 10.1016/0006-291x(84)90268-7. PubMed PMID: 6091633.

60. Fabbro D, Desnick RJ, Grabowski GA. Gaucher disease: genetic heterogeneity within and among the subtypes detected by immunoblotting. *Am J Hum Genet* (1987) 40(1):15-31. Epub 1987/01/01. PubMed PMID: 3812484; PubMed Central PMCID: PMCPMC1684010.

61. Barton NW, Furbish FS, Murray GJ, Garfield M, Brady RO. Therapeutic response to intravenous infusions of glucocerebrosidase in a patient with Gaucher disease. *Proc Natl Acad Sci U S A* (1990) 87(5):1913-6. Epub 1990/03/01. doi: 10.1073/pnas.87.5.1913. PubMed PMID: 2308952; PubMed Central PMCID: PMCPMC53594.

62. Ohashi T, Boggs S, Robbins P, Bahnson A, Patrene K, Wei FS, et al. Efficient transfer and sustained high expression of the human glucocerebrosidase gene in mice and their functional macrophages following transplantation of bone marrow transduced by a retroviral vector. *Proc Natl Acad Sci U S A* (1992) 89(23):11332-6. Epub 1992/12/01. doi: 10.1073/pnas.89.23.11332. PubMed PMID: 1454816; PubMed Central PMCID: PMCPMC50544.

63. Correll PH, Colilla S, Dave HP, Karlsson S. High levels of human glucocerebrosidase activity in macrophages of long-term reconstituted mice after retroviral infection of hematopoietic stem cells. *Blood* (1992) 80(2):331-6. Epub 1992/07/15. PubMed PMID: 1627794.

64. Ringden O, Groth CG, Erikson A, Backman L, Granqvist S, Mansson JE, et al. Long-term follow-up of the first successful bone marrow transplantation in Gaucher disease. *Transplantation* (1988) 46(1):66-70. Epub 1988/07/01. doi: 10.1097/00007890-198807000-00011. PubMed PMID: 3134756.

65. Nolta JA, Yu XJ, Bahner I, Kohn DB. Retroviral-mediated transfer of the human glucocerebrosidase gene into cultured Gaucher bone marrow. *J Clin Invest* (1992) 90(2):342-8. Epub 1992/08/01. doi: 10.1172/JCI115868. PubMed PMID: 1379609; PubMed Central PMCID: PMCPMC443108.

66. Xu L, Stahl SK, Dave HP, Schiffmann R, Correll PH, Kessler S, et al. Correction of the enzyme deficiency in hematopoietic cells of Gaucher patients using a clinically acceptable retroviral supernatant transduction protocol. *Exp Hematol* (1994) 22(2):223-30. Epub 1994/02/01. PubMed PMID: 8299741.

67. Hobbs JR, Jones KH, Shaw PJ, Lindsay I, Hancock M. Beneficial effect of pre-transplant splenectomy on displacement bone marrow transplantation for Gaucher's syndrome. *Lancet* (1987) 1(8542):1111-5. Epub 1987/05/16. doi: 10.1016/s0140-6736(87)91673-4. PubMed PMID: 2883444.

68. Correll PH, Fink JK, Brady RO, Perry LK, Karlsson S. Production of human glucocerebrosidase in mice after retroviral gene transfer into multipotential hematopoietic progenitor cells. *Proc Natl Acad Sci U S A* (1989) 86(22):8912-6. Epub 1989/11/01. doi: 10.1073/pnas.86.22.8912. PubMed PMID: 2573069; PubMed Central PMCID: PMCPMC298400.

69. Fink JK, Correll PH, Perry LK, Brady RO, Karlsson S. Correction of glucocerebrosidase deficiency after retroviral-mediated gene transfer into hematopoietic progenitor cells from patients with Gaucher disease. *Proc Natl Acad Sci U S A* (1990) 87(6):2334-8. Epub 1990/03/01. doi: 10.1073/pnas.87.6.2334. PubMed PMID: 2315324; PubMed Central PMCID: PMCPMC53681.

70. Karlsson S. Treatment of genetic defects in hematopoietic cell function by gene transfer. *Blood* (1991) 78(10):2481-92. Epub 1991/11/15. PubMed PMID: 1824245.

71. Jeckel D, Karrenbauer A, Burger KN, van Meer G, Wieland F. Glucosylceramide is synthesized at the cytosolic surface of various Golgi subfractions. *J Cell Biol* (1992) 117(2):259-67. Epub 1992/04/01. doi: 10.1083/jcb.117.2.259. PubMed PMID: 1532799; PubMed Central PMCID: PMCPMC2289419.

72. Futerman AH, Pagano RE. Determination of the intracellular sites and topology of glucosylceramide synthesis in rat liver. *Biochem J* (1991) 280 ( Pt 2):295-302. Epub 1991/12/01. doi: 10.1042/bj2800295. PubMed PMID: 1747103; PubMed Central PMCID: PMCPMC1130545.

73. Ichikawa S, Sakiyama H, Suzuki G, Hidari KI, Hirabayashi Y. Expression cloning of a cDNA for human ceramide glucosyltransferase that catalyzes the first glycosylation step of glycosphingolipid synthesis. *Proc Natl Acad Sci U S A* (1996) 93(10):4638-43. Epub 1996/05/14. PubMed PMID: 8643456; PubMed Central PMCID: PMC39331.

74. Yamashita T, Wada R, Sasaki T, Deng C, Bierfreund U, Sandhoff K, et al. A vital role for glycosphingolipid synthesis during development and differentiation. *Proc Natl Acad Sci U S A* (1999) 96(16):9142-7. Epub 1999/08/04. PubMed PMID: 10430909; PubMed Central PMCID: PMCPMC17746.

75. Futerman AH, Stieger B, Hubbard AL, Pagano RE. Sphingomyelin synthesis in rat liver occurs predominantly at the cis and medial cisternae of the Golgi apparatus. *J Biol Chem* (1990) 265(15):8650-7. Epub 1990/05/25. PubMed PMID: 2187869.

76. Ichikawa S, Hirabayashi Y. Glucosylceramide synthase and glycosphingolipid synthesis. *Trends Cell Biol* (1998) 8(5):198-202. Epub 1998/08/08. PubMed PMID: 9695839.

77. Lipsky NG, Pagano RE. Intracellular translocation of fluorescent sphingolipids in cultured fibroblasts: endogenously synthesized sphingomyelin and glucocerebroside analogues pass through the Golgi apparatus en route to the plasma membrane. *J Cell Biol* (1985) 100(1):27-34. Epub 1985/01/01. doi: 10.1083/jcb.100.1.27. PubMed PMID: 3965473; PubMed Central PMCID: PMCPMC2113465.

78. Schwarz A, Rapaport E, Hirschberg K, Futerman AH. A regulatory role for sphingolipids in neuronal growth. Inhibition of sphingolipid synthesis and degradation have opposite effects on axonal branching. *J Biol Chem* (1995) 270(18):10990-8. Epub 1995/05/05. doi: 10.1074/jbc.270.18.10990. PubMed PMID: 7738041.

79. Jeckel D, Karrenbauer A, Birk R, Schmidt RR, Wieland F. Sphingomyelin is synthesized in the cis Golgi. *FEBS Lett* (1990) 261(1):155-7. Epub 1990/02/12. doi: 10.1016/0014-5793(90)80659-7. PubMed PMID: 2155131.

80. Coste H, Martel MB, Got R. Topology of glucosylceramide synthesis in Golgi membranes from porcine submaxillary glands. *Biochim Biophys Acta* (1986) 858(1):6-12. Epub 1986/06/13. doi: 10.1016/0005-2736(86)90285-3. PubMed PMID: 2939881.

81. Steet RA, Chung S, Wustman B, Powe A, Do H, Kornfeld SA. The iminosugar isofagomine increases the activity of N370S mutant acid beta-glucosidase in Gaucher fibroblasts by several mechanisms. *Proc Natl Acad Sci U S A* (2006) 103(37):13813-8. Epub 2006/09/02. doi: 0605928103 [pii]

10.1073/pnas.0605928103. PubMed PMID: 16945909; PubMed Central PMCID: PMC1564243.

82. Sawkar AR, Cheng WC, Beutler E, Wong CH, Balch WE, Kelly JW. Chemical chaperones increase the cellular activity of N370S beta -glucosidase: a therapeutic strategy for Gaucher disease. *Proc Natl Acad Sci U S A* (2002) 99(24):15428-33. Epub 2002/11/16. doi: 10.1073/pnas.192582899. PubMed PMID: 12434014; PubMed Central PMCID: PMCPMC137733.

83. Ron I, Horowitz M. ER retention and degradation as the molecular basis underlying Gaucher disease heterogeneity. *Hum Mol Genet* (2005) 14(16):2387-98. Epub 2005/07/08. doi: ddi240 [pii]

10.1093/hmg/ddi240. PubMed PMID: 16000318.

84. Dvir H, Harel M, McCarthy AA, Toker L, Silman I, Futerman AH, et al. X-ray structure of human acid-beta-glucosidase, the defective enzyme in Gaucher disease. *EMBO Rep* (2003) 4(7):704-9. Epub 2003/06/07. doi: 10.1038/sj.embor.embor873. PubMed PMID: 12792654; PubMed Central PMCID: PMCPMC1326319.

85. Maegawa GH, Tropak MB, Buttner JD, Rigat BA, Fuller M, Pandit D, et al. Identification and characterization of ambroxol as an enzyme enhancement agent for Gaucher disease. *J Biol Chem* (2009) 284(35):23502-16. Epub 2009/07/07. doi: 10.1074/jbc.M109.012393. PubMed PMID: 19578116; PubMed Central PMCID: PMCPMC2749124.

86. Sawkar AR, Adamski-Werner SL, Cheng WC, Wong CH, Beutler E, Zimmer KP, et al. Gaucher disease-associated glucocerebrosidases show mutation-dependent chemical chaperoning profiles. *Chem Biol* (2005) 12(11):1235-44. Epub 2005/11/22. doi: 10.1016/j.chembiol.2005.09.007. PubMed PMID: 16298303.

87. Lieberman RL, Wustman BA, Huertas P, Powe AC, Jr., Pine CW, Khanna R, et al. Structure of acid beta-glucosidase with pharmacological chaperone provides insight into Gaucher disease. *Nat Chem Biol* (2007) 3(2):101-7. Epub 2006/12/26. doi: nchembio850 [pii]

10.1038/nchembio850. PubMed PMID: 17187079.

88. Jmoudiak M, Futerman AH. Gaucher disease: pathological mechanisms and modern management. *Br J Haematol* (2005) 129(2):178-88. Epub 2005/04/09. doi: 10.1111/j.1365-2141.2004.05351.x. PubMed PMID: 15813845.

89. Khanna R, Benjamin ER, Pellegrino L, Schilling A, Rigat BA, Soska R, et al. The pharmacological chaperone isofagomine increases the activity of the Gaucher disease L444P mutant form of beta-glucosidase. *FEBS J* (2010) 277(7):1618-38. Epub 2010/02/13. doi: EJB7588 [pii]

10.1111/j.1742-4658.2010.07588.x. PubMed PMID: 20148966; PubMed Central PMCID: PMC2874831.

90. Shaaltiel Y, Bartfeld D, Hashmueli S, Baum G, Brill-Almon E, Galili G, et al. Production of glucocerebrosidase with terminal mannose glycans for enzyme replacement therapy of Gaucher's disease using a plant cell system. *Plant Biotechnol J* (2007) 5(5):579-90. Epub 2007/05/26. doi: 10.1111/j.1467-7652.2007.00263.x. PubMed PMID: 17524049.

91. Glew RH, Basu A, LaMarco KL, Prence EM. Mammalian glucocerebrosidase: implications for Gaucher's disease. *Lab Invest* (1988) 58(1):5-25. Epub 1988/01/01. PubMed PMID: 3275832.

92. O'Brien JS, Kretz KA, Dewji N, Wenger DA, Esch F, Fluharty AL. Coding of two sphingolipid activator proteins (SAP-1 and SAP-2) by same genetic locus. *Science* (1988) 241(4869):1098-101. Epub 1988/08/26. doi: 10.1126/science.2842863. PubMed PMID: 2842863.

93. Morimoto S, Kishimoto Y, Tomich J, Weiler S, Ohashi T, Barranger JA, et al. Interaction of saposins, acidic lipids, and glucosylceramidase. *J Biol Chem* (1990) 265(4):1933-7. Epub 1990/02/05. PubMed PMID: 2298731.

94. Morimoto S, Martin BM, Yamamoto Y, Kretz KA, O'Brien JS, Kishimoto Y. Saposin A: second cerebrosidase activator protein. *Proc Natl Acad Sci U S A* (1989) 86(9):3389-93. Epub 1989/05/01. doi: 10.1073/pnas.86.9.3389. PubMed PMID: 2717620; PubMed Central PMCID: PMCPMC287138.

95. Schnabel D, Schroder M, Sandhoff K. Mutation in the sphingolipid activator protein 2 in a patient with a variant of Gaucher disease. *FEBS Lett* (1991) 284(1):57-9. Epub 1991/06/17. doi: 10.1016/0014-5793(91)80760-z. PubMed PMID: 2060627.

96. Christomanou H, Aignesberger A, Linke RP. Immunochemical characterization of two activator proteins stimulating enzymic sphingomyelin degradation in vitro. Absence of one of them in a human Gaucher disease variant. *Biol Chem Hoppe Seyler* (1986) 367(9):879-90. Epub 1986/09/01. doi: 10.1515/bchm3.1986.367.2.879. PubMed PMID: 3024666.

97. Klein A, Henseler M, Klein C, Suzuki K, Harzer K, Sandhoff K. Sphingolipid activator protein D (sap-D) stimulates the lysosomal degradation of ceramide in vivo. *Biochem Biophys Res Commun* (1994) 200(3):1440-8. Epub 1994/05/16. doi: 10.1006/bbrc.1994.1612. PubMed PMID: 8185598.

98. Berent SL, Radin NS. Mechanism of activation of glucocerebrosidase by co-beta-glucosidase (glucosidase activator protein). *Biochim Biophys Acta* (1981) 664(3):572-82. Epub 1981/06/23. doi: 10.1016/0005-2760(81)90134-x. PubMed PMID: 6268176.

99. Kleinschmidt T, Christomanou H, Braunitzer G. Complete amino-acid sequence and carbohydrate content of the naturally occurring glucosylceramide activator protein (A1 activator) absent from a new human Gaucher disease variant. *Biol Chem Hoppe Seyler* (1987) 368(12):1571-8. Epub 1987/12/01. doi: 10.1515/bchm3.1987.368.2.1571. PubMed PMID: 3442600.

100. Iyer SS, Berent SL, Radin NS. The cohydrolases in human spleen that stimulate glucosyl ceramide beta-glucosidase. *Biochim Biophys Acta* (1983) 748(1):1-7. Epub 1983/10/17. doi: 10.1016/0167-4838(83)90020-1. PubMed PMID: 6615847.
